# Sport-related Concussion Can be Prevented by Injury Prevention Program: A Systematic Review and Meta-analysis of Prospective, Controlled Studies

**DOI:** 10.1186/s40798-025-00936-4

**Published:** 2025-11-21

**Authors:** Yan-Long Chen, Tsung-Yeh Chou, Ming-Chih Sung, Yu-Lun Huang

**Affiliations:** 1 Department of Rehabilitation Medicine, Ningbo No. 2 Hospital, Ningbo, China; 2 Faculty of Sports Science, Ningbo University, Ningbo, China; 3 Department of Orthopedic Surgery, University of Texas Health Science Center at Houston, Houston, TX USA; 4 School of Kinesiology, California State University, Angeles, CA USA; 5 Department of Physical Education and Sport Sciences, National Taiwan Normal University, Taipei City, Taiwan

**Keywords:** Mild traumatic brain injury, Training, Education, Adolescents

## Abstract

**Background:**

Sport-related concussions (SRCs) have emerged as a global health concern in sports medicine. Effective injury prevention programs have the potential to reduce the risk of SRCs, but, their efficacy remains inconclusive. Therefore, the aim of this systematic review and meta-analysis was to evaluate the efficacy of injury prevention programs in preventing SRCs and to examine whether different intervention types (physical- vs. educational-based) and athletic exposure context (practice vs. match) influenced the efficacy of interventions.

**Methods:**

Six databases (PubMed, CINAHL, MEDLINE, SPORTDiscus, Scopus, and Embase databases) were searched in March 2024. Studies were included if (1) the physical training or educational intervention aimed to prevent SRC, (2) the incidence rate (IR) or other outcome data sufficient to calculate the IR for both the intervention and control groups were reported, and (3) the study employed a prospective design.

**Results:**

A total of eight studies were included in the analysis, involving 2571 participants (intervention group: *n* = 1281; control group: *n* = 1290). Current injury prevention programs primarily targeted male athletes in rugby, American Football, and soccer. The analysis revealed a significant reduction in SRC rate among athletes who received injury prevention programs (incidence rate ratio = 0.66, 95% CI [0.50–0.85], *p* = .002). The moderator analysis indicated that neither the type of intervention nor the exposure context significantly influenced the efficacy of the injury prevention programs.

**Conclusions:**

SRC prevention programs provided a significant protection effect, reducing injury rates by 34%. Importantly, their efficacy remains consistent across both physical training and educational-based programs, as well as in various exposure contexts. However, further prospective studies are needed to develop injury prevention protocols specifically for females and to investigate factors that may influence the efficacy of these interventions to enhance the prevention of SRC across various sports.

## Key Points

- SRC injury prevention programs can reduce the incidence rate of SRC by 34%.
- The efficacy of injury prevention programs is unaffected by whether the intervention is physical training- or education-based, or by the context of athletic exposure (practice or match).
- Current injury prevention programs predominantly target male athletes in rugby, American Football, and soccer, highlighting the need for more research focused on female athletes and other sports.

## Background

For nearly two decades, sport-related concussions (SRCs) have been recognized as a significant global health concern within the field of sports medicine. In the United States, approximately 1.6 million to 3.8 million SRCs occur annually [1], leading to an average direct healthcare cost of approximately US$800 per case between 2017 and 2020 [2]. SRC not only increases the risk of subsequent concussions but also elevates the potential likelihood of other health issues, including a greater risk of subsequent musculoskeletal injuries upon return to play [3–5], as well as neurological disorders (e.g. dementia) and neurodegenerative diseases (e.g. Parkinson’s disease) [6–8]. To summarize, given the prevalence, medical burden, and associated deteriorated health consequences of SRC, prevention of SRC should be given utmost priority.

Injury prevention programs generally target modifiable risk factors [9]. Most SRC injury prevention programs focus on training interventions aimed at addressing modifiable intrinsic risk factors [10], such as weak neck strength [11] and improper tackling techniques [12] in the context of SRC. Previous systematic reviews have provided preliminary insights into the effects of injury prevention programs [13–16]. For instance, Pankow et al. [14] reviewed three studies on educational-based interventions and concluded that the Heads Up Football (HUF) program reduced SRC incidence rates by 32% among high school football players. Eliason et al. [13] presented evidence indicating that implementing a neuromuscular warm-up program in rugby can lower SRC incidence rates by up to 60%. Two earlier reviews published in 2017 [15, 16] included a limited number of studies and were unable to draw definitive conclusions about the efficacy of education and training interventions. However, these earlier reviews have notable limitations: (1) the reviews did not perform a meta-analysis of relevant training strategies for SRC risk reduction, likely due to a lack of sufficient data at the time the review was undertaken [13–16]; (2) they did not encompass other key intervention programs related to SRC prevention, such as neck strength training [17, 18]; (3) the studies included in these reviews were not exclusively prospective controlled studies [13, 14], which limits the ability to establish a clear cause-and-effect relationship between the interventions and their efficacy in preventing SRC. Given the growing body of evidence, a meta-analysis of prospective controlled studies is warranted to synthesize effect sizes across studies, address the aforementioned limitations, and provide a more robust and generalizable understanding of the efficacy of injury prevention programs in reducing SRC incidence [19].

Additionally, the efficacy of injury prevention programs for preventing SRCs may vary depending on the types of intervention and the context of athletic exposure. Common SRCs injury prevention interventions can be categorized into physical training-based and educational-based. Physical training-based interventions generally include a combination of strength, balance, proprioceptive, and plyometric exercises, designed to enhance physical fitness, thereby improving athletic performance and reducing injury risk [17, 20–23]. In contrast, educational-based programs primarily focus on imparting knowledge and sport-related skills to prevent or mitigate SRC, as exemplified by initiatives such as the HUF program [24–26]. Given the distinct methodologies associated with these intervention types, the nature of the intervention may serve as a critical factor influencing its efficacy [27]. Additionally, the efficacy of an intervention may also vary depending on the context of athletic exposure. For instance, in soccer (football), overall injury rates are higher during matches compared to practice sessions [28]. The FIFA 11 + warm-up program has demonstrated differing efficacy in these two contexts of athletic exposure, reducing soccer-related injury by 40% during matches and by 30% during practice sessions [29]. Given the disparities in SRC incidence rates between matches and practices [30–32], it is likely that SRC injury prevention programs will exhibit varying efficacy across athletic exposures.

The primary aim of this systematic review and meta-analysis was to evaluate the efficacy of injury prevention programs in preventing SRC. Additionally, our study examined whether the efficacy of these intervention programs varied based on intervention type and athletic exposure context. We hypothesized that: (1) SRC injury prevention programs can effectively reduce the risk of SRC, and (2) the efficacy of these intervention programs varies depending on the type of intervention and the context of athletic exposure.

## Methods

This systematic review and meta-analysis was pre-registered at the International Prospective Register of Systematic Reviews (CRD42022375416), and the Preferred Reporting Items for Systematic Reviews and Meta-Analyses guidelines were followed [33].

### Searches

A systematic search was performed using the PubMed, CINAHL, MEDLINE, SPORTDiscus, Scopus, and Embase databases from January 2002 to March 2024. The keywords were divided into three categories (1) concussion, (2) population, (3) training, and their synonyms were used to search for related study articles (Table 1). The keywords and their synonyms within each category (concussion/population/intervention) were combined using the Boolean operator ‘‘OR,’’ and all three categories were combined to form one search using the Boolean operator ‘‘AND.’’ In addition, the reference lists of previous meta-analyses on relevant topic were manually searched for additional studies not already identified.

**Table 1:** Search terms

Category	Search terms
Concussion	“concussion”
	**OR** “mild traumatic brain injur*”
	**OR** “mTBI”
	**OR** “brain injur*”
	**OR** “brain concussion”
	**OR** “head injur*”
**AND**	
Population	“sport*”
	**OR** “athlet*”
**AND**	
Intervention	“prevent* **AND** control*”
	**OR** “injur* **AND** prevent*”
	**OR** “injur* **AND** avoidance*”
	**OR** “educat*”
	**OR** “train*”

### Study Inclusion and Exclusion Criteria

Studies were selected throughout the screening process if they met the following criteria: (1) the physical or educational training aimed to prevent SRC, (2) the incidence rate (IR) or other outcome data that made it possible to calculate the IR for both the intervention and control groups were reported, and (3) the study design was a prospective study. The operational definition of physical training is any training designed to warm-up or strengthen the body in order to enhance athletic performance and prevent injury [10]. Educational intervention is defined as one focused on acquiring knowledge and skills to prevent or mitigate injuries, such as learning proper tackling techniques and concussion knowledge [24–26]. Exclusion criteria involved: (1) the following types of articles: review articles, abstracts, speeches, editorials, comments, non-peer-reviewed articles or case studies, (2) articles not written in English.

### Potential Effect Modifiers and Reasons for Heterogeneity

After removing duplicates, two independent reviewers (YLC and TYC) conducted the title and abstract screening with the predetermined eligibility criteria. Articles that were unclear in the initial title and abstract screens were included in a full text screen. If the two authors agreed that the study was relevant, the full text was subsequently reviewed. All disagreements in full text screening were settled by a third reviewer (YLH). Cohen kappa coefficient (k) was used to calculate inter-rater agreement between the two reviewers. Magnitude guidelines have been suggested by Landis and Koch, who characterized values less than 0 as indicating no agreement, 0–0.20.20 as slight agreement, 0.21–0.40 as fair agreement, 0.41–0.60 as moderate agreement, 0.61–0.80 as substantial agreement, and 0.81–1.81 as almost perfect agreement [34].

### Study Quality Assessment

The quality of the included studies was assessed using the National Institutes of Health (NIH) Study Quality Assessment Tool for Controlled Intervention Studies [35]. This tool includes 14 items that evaluate key aspects such as randomization, blinding, sample size, baseline comparability, adherence, outcome measurement, and statistical analysis. Two reviewers (YLC and TYC) independently conducted the quality assessments and assigned an overall quality rating of “Good”, “Fair”, or “Poor”. Disagreements were resolved through consensus. If necessary, a third author was consulted to achieve consensus.

### Data Extraction Strategy

Data extraction was completed by the first author YLC using a structured form, and then the second author TYC verified the extracted data independently to ensure accuracy. The extracted data included the following: author, type of training, study location, type of sport, participant characteristics, comparison groups (intervention and control descriptions), training characteristics (duration, intensity, time, compliance and dropout rate), and outcome measures (incidence rate ratio [IRR] or other relevant findings such as IR).

### Data Synthesis and Presentation

The IRR of SRCs from selected studies were aggregated using R Studio (version 2023.12.0–369.0), with the ‘meta’ and ‘metafor’ packages. If the IRR was not available in selected studies, it was calculated by dividing the incidence rate in the intervention group by the incidence rate in the control group [26]. The incidence rates were determined by dividing the number of SRC events by the athlete exposures (AEs) for each group. Specifically, the IR for the intervention group was calculated as the number of SRCs in the intervention group divided by the AEs in the intervention group, and the incidence rate for the control group was calculated as the number of SRCs in the control group divided by the AEs in the control group. Acknowledging the inherent diversity among the studies, such as variations in intervention programs, a random-effects model was used to determine the summarized IRR. This model choice, as suggested by Borenstein et al. [27], ensured a more balanced distribution of study weights.

To evaluate the statistical heterogeneity among the studies, Cochran’s Q and the I² statistic were used. Heterogeneity refers to the variation of outcomes observed across the studies. A Cochran’s Q statistic with a p-value less than 0.05 was indicative of significant heterogeneity. The I² values offered a quantitative measure of this heterogeneity: values of ≤ 25% were categorized as low, 50% as moderate, and ≥ 75% as high heterogeneity. Potential publication bias was examined using Egger’s regression test, complemented by the analysis of a funnel plot. An Egger’s regression test p-value below 0.05 was considered indicative of notable publication bias. The funnel plot, which plots standard error against effect size, served as a visual tool for assessing bias. A symmetrical plot was interpreted as indicative of an unbiased range of effect sizes, whereas asymmetry suggested the possibility of publication bias.

Further, moderator analyses were conducted to investigate factors potentially affecting the efficacy of interventions in preventing SRC. The factors included (1) intervention type (physical- vs. educational-based) and (2) context of athletic exposure (practice vs. match). It is worth noting that one data point [18] was excluded from the ‘context of participations’ moderator analysis because it combined both match and practice contexts. For a variable to be included in the moderator analyses, at least two data points were required to meet the criteria for the variable of interest [27]. The selection of these particular factors was based on their demonstrated relevance to SRC incidence rates, as identified in prior research [20, 24, 31].

## Results

### Search Results

**Fig. 1:**
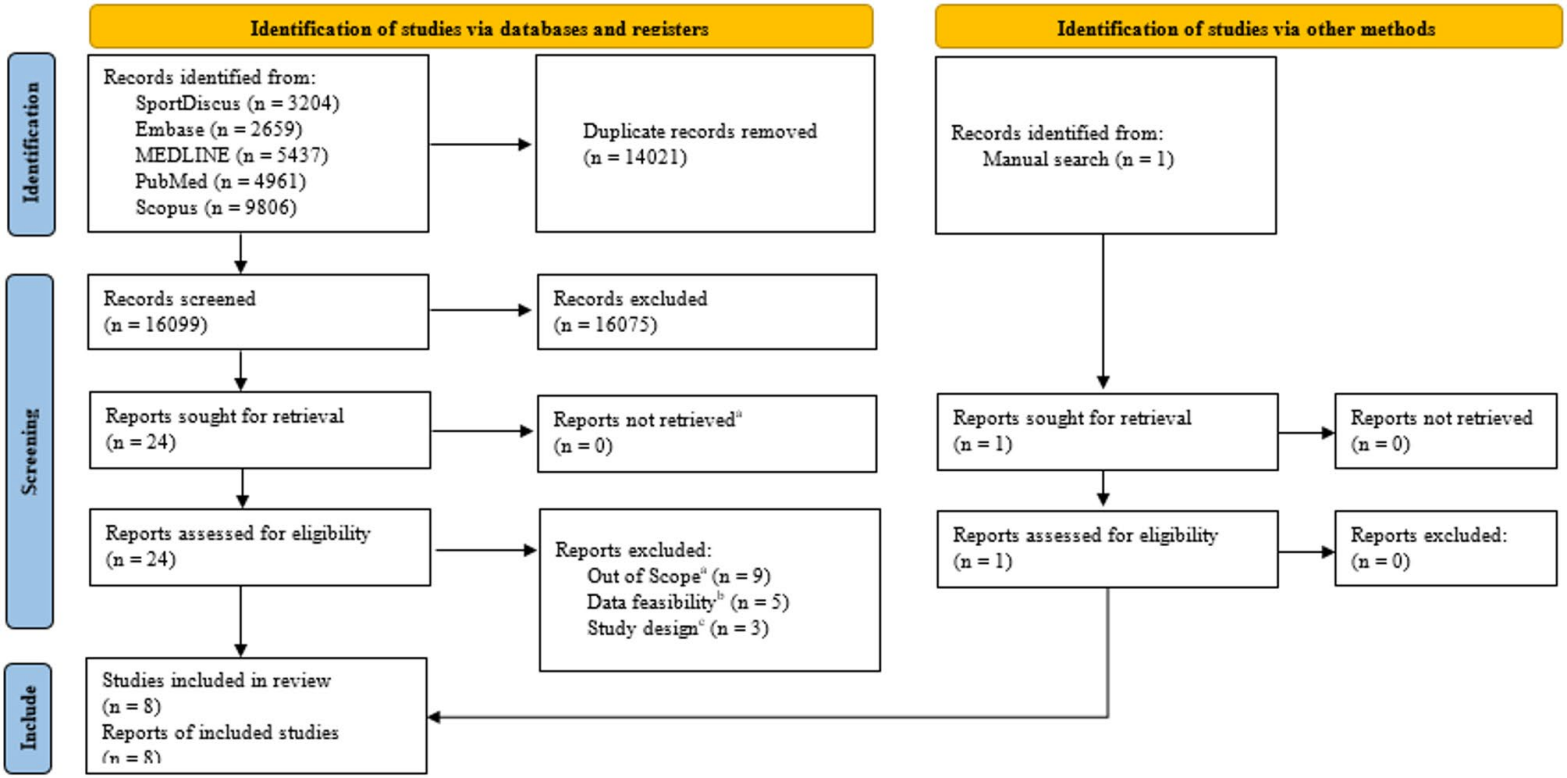
Preferred Reporting Items for Systematic Reviews and Meta-Analyses flowdiagram for study selection. ^a^Other intervention. ^b^Could not calculate incidence rate. Non-prospective study

Our database search yielded a total of 30,120 articles. After eliminating duplicates, the yield was lowered to 16,099. Among these, 10,675 studies were excluded after assessing the titles and abstracts (percent agreement = 89.6%, κ = 0.57), while 24 studies were retained for full-text review to determine eligibility. One additional study was identified through manual search during the full-text review. The screening of full text resulted in the removal of 17 studies for the following reasons: nine studies determined the interventions were not relevant to physical or educational approaches to preventing SRC, five studies had insufficient data to calculate incidence rates, and three studies were not prospective research. In the Ultimately, eight studies met the criteria and were included in the meta-analysis (percent agreement = 85%, κ = 0.66) (Fig. 1).

### Study Characteristics

The total number of participants was 2571 (intervention group (*n* = 1281); control group (*n* = 1290)), and details of the eight included studies are provided in Table 2. Exposure and injury data for all training intervention are provided in Table 3. In terms of study design, three studies were cluster randomized controlled trials [20, 22, 36], three were cohort studies [18, 24, 26], one was a quasi-experimental study [21], and one was a pre–post intervention study [17]. Regarding SRC diagnosis, five of the eight studies did not report specific criteria for diagnosing SRC [17, 20–22, 36]. Two studies stated that SRCs were determined by certified athletic trainers [24, 26], while one study relied on self-reports from players [18]. Three studies were conducted in the United Kingdom [20–22], two were conducted in the United States [24, 26], two were conducted in Australia [17, 18] and one was conducted in Kosovo [36]. The study included three sports: rugby [17, 20–22], American Football [24, 26], and soccer [18, 36]. The majority of the studies (*n* = 7) included only male participants [17, 20–22, 24, 26, 36], and one study included both sexes [18]. Six studies focused on physical training-based injury prevention interventions [17, 18, 20–22, 36], while two studies implemented educational-based interventions [24, 26]. Three of the studies recorded SRC incidence only during matches [20–22], one only during practice [24], three in both contexts [17, 26, 36], and one mixed practice and match contexts SRC incidence [18].

**Table 2:** Study characteristics

Study	Type of Int	Study Location	Type of Sport	Participant Age, y mean (SD)	Sex, (M/F)	Sample Size (*n*)	Int method	Int time (1 = pre-season, 2 = in-, 3 = post-)	Int Duration	Training Time	Follow-up Duration	Compliance	Dropouts in the Int Group, %
Attwood et al. (2018) [20]	Physical	UK	Rugby	Int: 26.1 (5.7) Con: 25.0 (5.5)	M	Int: 682 Con: 673	Warm up program	1 & 2	42-week	20–25 min, 2×/week for training sessions; 1×/week for pre-match	One season	Int: 85%* Con: 83%*	49% (base on group)
Barden et al. (2022) [21]		UK	Rugby	Int: 15.9 (1.6) Con: 15.0 (2.0)	M	Int: 412 Con: 247	Warm up program	2	One season	15 min, 3×/week	One season	Int: 86%^†^ Con: 80%^†^	65% (base on group)
Gillies et al. (2022) [17]		Australia	Rugby	Forward group: 24.08 (3.94) Back group: 22.51 (2.95)	M	Int: 39 Con: 39	Neck muscle training program	1 & 2	27-week	NR, 3×/week for phase1-2, 2×/week for phase3	One season	NR	0%
Hislop et al. (2017) [22]		UK	Rugby	Int: 16.0 (1.2) Con: 15.9 (1.1)	M	Int: 1325 Con: 1127	Warm up program	1 & 2	13 or 14-week	20 min, 3×/week	One season	Int: 69%^‡^ Con: 83%^‡^	19%
Obërtinca (2024) [36]		Kosovo	Soccer	Int: 15.2 (1.6) Con: 15.3 (1.6)	M	Int: 524 Con: 503	Exercise-based program	1 & 2	One season	15–20 min, least 2×/week	One season	Int: 72.2%^§^ Con: NR	18%
Peek et al. (2023) [18]		Australia	Soccer	NR	M&F	Int: 146 Con: 218	Neck muscle training program	2	One season	3 min, 2–3×/week	One season	NR	0%
Kerr et al. (2015) [26]	Education	USA	American Football	Int: 10.94 (1.82) Con: 10.56 (1.90)	M	Int: 663 Con: 704	HUF program	1	NR	NR	One season	NR	NR
Shanley et al. (2021) [24]		USA	American Football	Int: 15.1 (1.4) Con: 14.8 (1.6)	M	Int: 1818 Con: 696	HUF program	1 & 2	One season	NR	One season	NR	NR

HUF: heads up Heads Up Football; NR: not reported; Int: intervention; Con: control

* Compliance was reported as the median session attendance rate

^†^ Compliance was reported as the mean session attendance

^‡^ Compliance was reported as the proportion of program components completed

^§^ Compliance was reported as the proportion of training sessions in which the intervention was implemented

**Table 3:** Exposure and injury data for all training intervention

Study	Intervention	Participants analyzed	Context	Exposure hours	SRC number
Attwood et al. (2018) [20]	Physical training	Int: 682; Con: 673	Match	Int: 9900; Con: 9660	Int: 12; Con: 33
Barden et al. (2022) [21]		Int: 412; Con: 247	Match	Int: 3116; Con: 1524	Int: 24; Con: 13
Gillies et al. (2022) [17] ^†^		Int: 39; Con: 39	Practice Match	Int: 1905; Con: 4287 Int: 180; Con: 359	Int:1; Con: 3 Int:1; Con: 4
Hislop et al. (2017) [22]		Int: 1325; Con: 1127	Match	Int: 9083; Con: 6855	Int: 51; Con: 54
Obërtinca (2024) [36] ^†^		Int: 524; Con: 503	Practice match	Int: 44,437; Con: 44,272 Int: 9017; Con: 8666	Int:1; Con: 1 Int:2; Con: 2
Peek et al. (2023) [18] *		Int: 146; Con: 218	Practice & match	Int: 18,011; Con: 27,485	Int: 2; Con: 13
Kerr et al. (2015) [26] ^‡§^	Educational training	Int: 663; Con: 704	Practice Match	Int: 15,500; Con: 20,560 Int: 4000; Con: 6850	Int: 10; Con: 12 Int: 6; Con: 10
Shanley et al. (2021) [24] ^§^		Int: 1818; Con: 696	Practice	Int: 1,413,043; Con: 386,447	Int: 37; Con: 19

* Data cannot be separated into practice and game context

^†^ Exposure hours and SRC number obtained by contacting the authors

^‡^ Exposure hours calculated based on incidence rate

^§^ Athletic exposure was defined as an athlete participating in 1 game or 1 practice

### Study Quality Assessment

The average score of the studies was 7 (5–10), suggesting an overall fair methodological quality. Details explaining the scoring for each criterion can be found in Table 4. The primary methodological limitations were related to randomization, baseline comparability, and the blinding of participants, therapists, and assessors.

**Table 4:** NIH quality assessment tool for controlled intervention studies

Author	1	2	3	4	5	6	7	8	9	10	11	12	13	14	Score
Attwood et al. (2018) [20]	Yes	Yes	No	Yes	Yes	No	No	Yes	Yes	Yes	Yes	CD	Yes	Yes	10
Barden et al. (2022) [21]	No	No	No	Yes	No	No	No	NR	Yes	NR	Yes	Yes	Yes	No	5
Gillies et al. (2022) [17]	No	No	No	No	No	Yes	Yes	NA	NR	NA	Yes	NR	Yes	Yes	5
Hislop et al. (2017) [22]	Yes	Yes	Yes	Yes	No	No	No	Yes	No	No	Yes	Yes	Yes	Yes	9
Kerr et al. (2015) [26]	No	No	No	No	No	No	Yes	Yes	NR	NR	Yes	CD	Yes	Yes	5
Obërtinca (2024) [36]	Yes	Yes	Yes	No	No	No	Yes	Yes	No	Yes	Yes	Yes	Yes	Yes	10
Peek et al. (2023) [18]	No	No	No	No	No	No	Yes	NR	Yes	Yes	Yes	Yes	Yes	Yes	7
Shanley et al. (2021) [24]	No	No	No	No	No	No	Yes	Yes	NR	NR	Yes	NR	Yes	Yes	5

(1) Randomization described; (2) Adequate randomization method; (3) Concealed allocation; (4) Blinding of participants; (5) Blinding of providers; 6 Baseline comparability; 7. ≤20% drop-out rate; 8. ≤15% difference in drop-out rates between groups; 9. High intervention adherence; 10. Similar in other treatments received; 11. Valid and reliable outcome measures; 12. Adequate sample size; 13. Pre-specified outcomes or subgroup analyses reported; 14. Intention-to-treat analysis. CD, cannot determine; NA, not applicable; NR, not reported

### Publication Bias

**Fig. 2:**
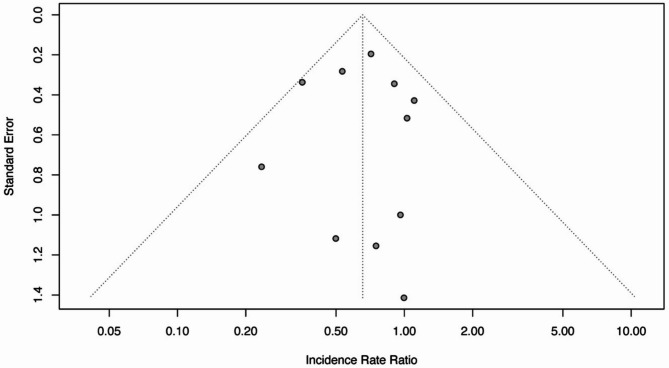
Funnel plot for visual inspection of publication bias

After visually inspecting the funnel plot of the included studies, it was observed that there was symmetry, as shown in Fig. 2, indicating no publication bias. This observation was further supported by the Egger’s regression test, which revealed no statistically significant evidence of publication bias (*p* =.97) among the included studies. Both tests’ results indicated the absence of publication bias among the included studies.

### Overall Intervention Effects

**Fig. 3:**
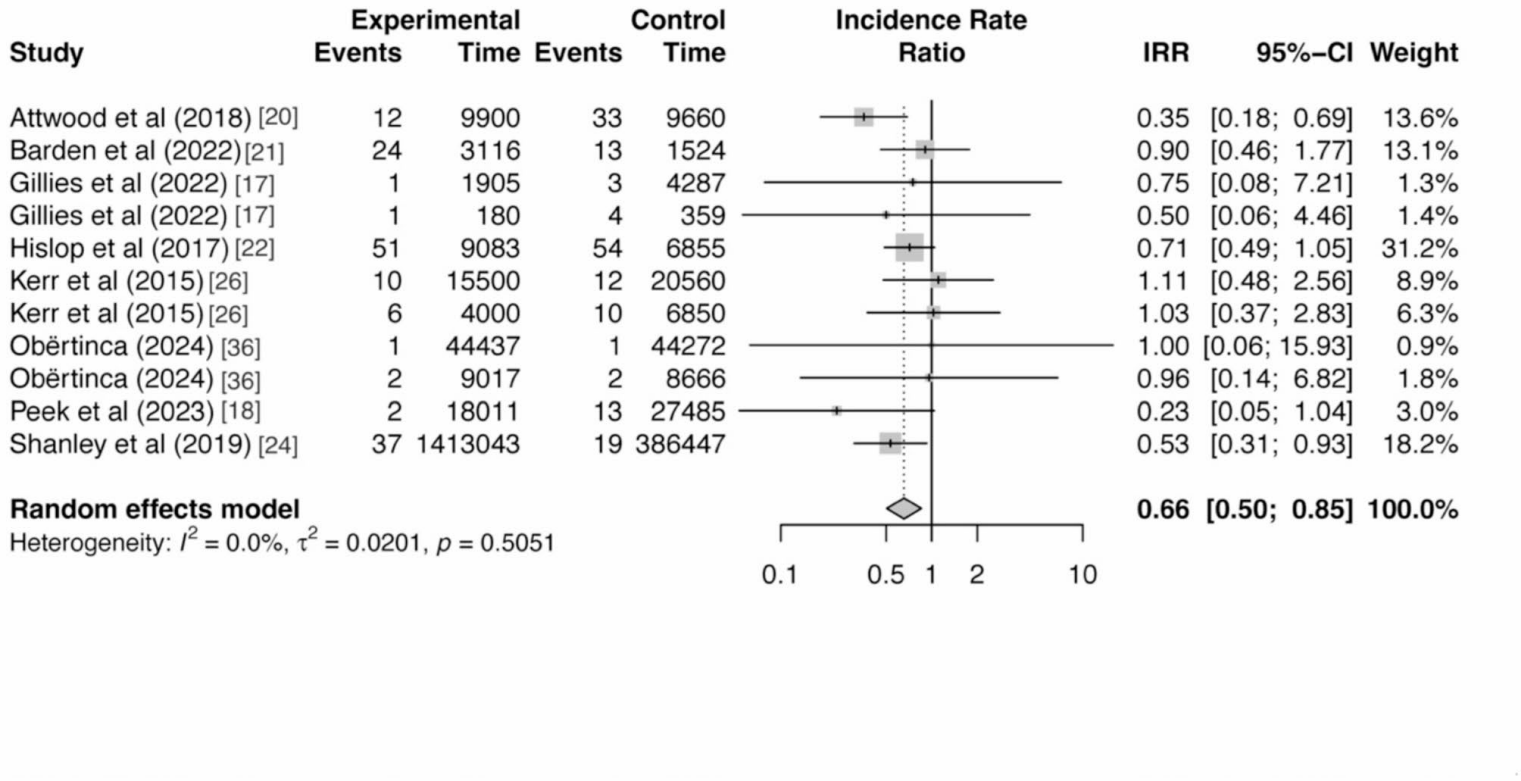
Forest plot for every study included in this analysis

Our results showed a significant decrease in the incidence rate of SRC when athletes participated in intervention programs (IRR = 0.66, 95% CI [0.50–0.85], *p* =.002). Athletes who received the intervention had a 34% lower incidence rate of SRC compared to those who did not receive the intervention. In addition, the result of Cochran’s *Q* test and *I*^*2*^ statistics were not statistically significant (*Q* = 9.29, *df* = 10, *I*^*2*^ = 0, *p* =.51), indicating that there was no heterogeneity among the included studies. The forest plot and the distribution of effect sizes from the included studies under the random-effects model are presented in Fig. 3.

### Moderator Analysis

**Fig. 4:**
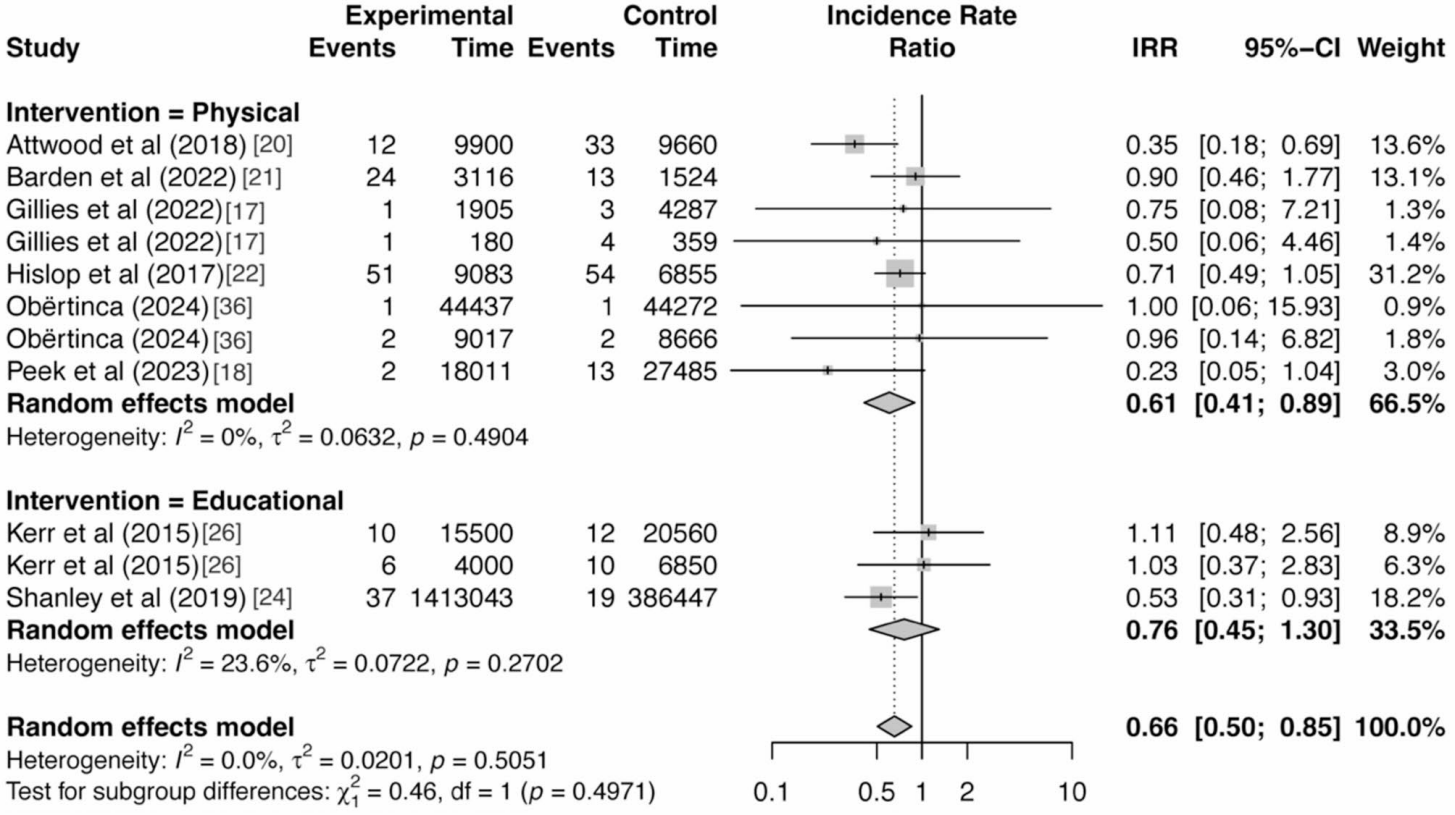
Forest plot of moderator analysis by intervention type

**Fig. 5:**
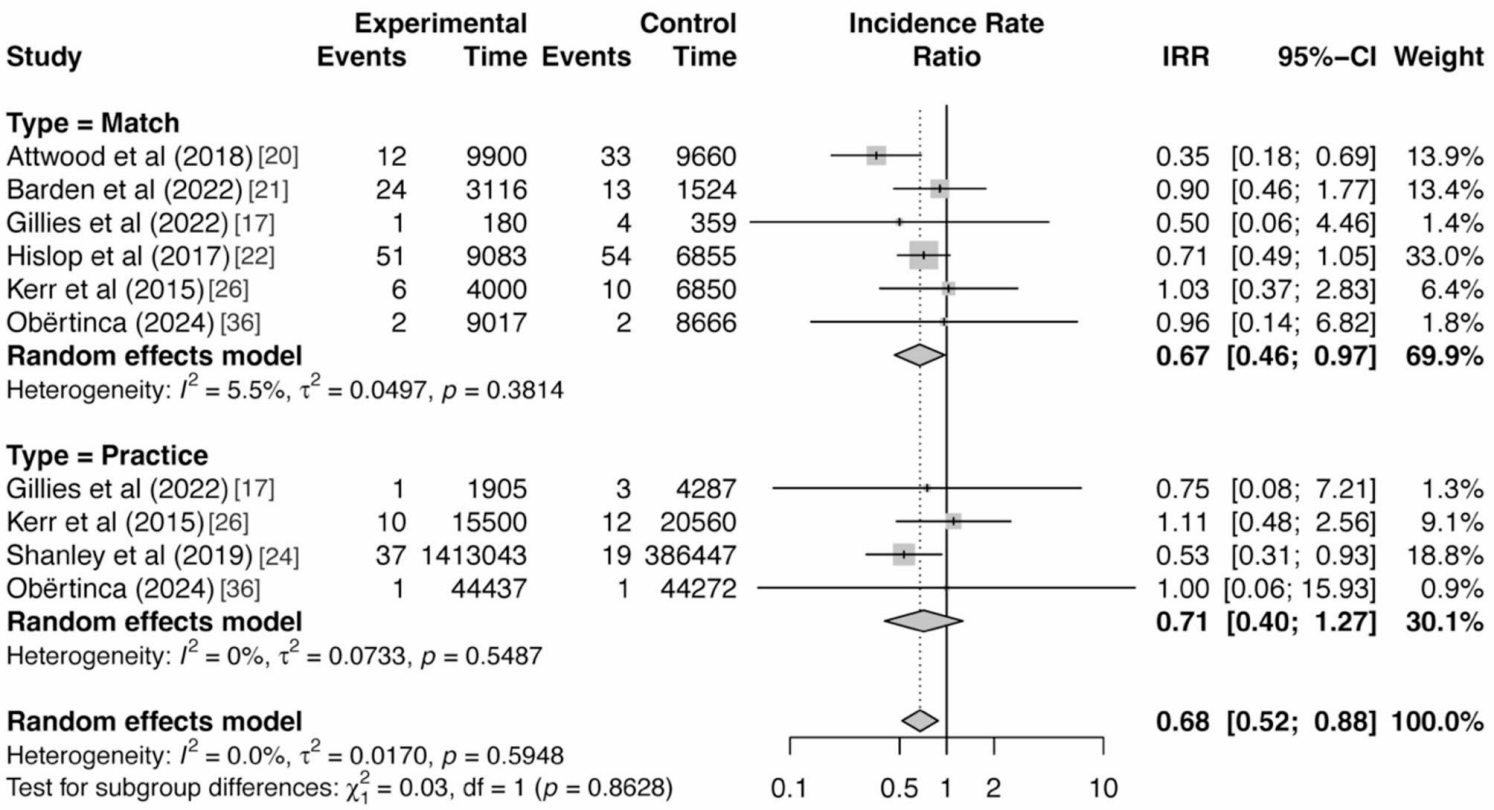
Forest plot of moderator analysis by athletic exposure context

Even though heterogeneity was not detected among the included studies, as previous literature suggested, methodological differences could influence the effect of interventions on SRC outcomes [27]. Therefore, moderator analyses were performed to investigate the potential impact of intervention type (physical- vs. educational-based) and context of athletic exposure (practice vs. match) on the overall effect. No statistical significance was found for either of the moderator variables, indicating that neither intervention type nor athletic exposure context had a significant influence on the overall effect of the interventions, as displayed in Table 5. The forest plots for the moderator analysis are provided in Figs. 4 and 5.

**Table 5:** Moderator analysis of intervention type and exposure context

Moderator	Effect Size				Heterogeneity		
	k	IRR	95% CI		Q	df	*p*
**Training Type**					0.46	1	0.50
Physical training	8	0.61	[0.42, 0.89]				
Educational intervention	3	0.76	[0.45, 1.30]				
**Athletic Exposure Context**					0.03	1	0.86
Match	6	0.67	[0.46, 0.97]				
Practice	4	0.71	[0.40, 1.27]				

Abbreviations: *k* = Number of data points; *IRR* = Incidence rate ratio; 95% CI = 95% confidence intervals; *Q* = Cochran’s *Q* statistic; *df* = degrees of freedom of *Q* statistic; *p* = p-value

## Discussion

The current meta-analysis investigated the efficacy of SRC injury prevention programs in reducing the incidence rates of SRC, while also exploring whether their efficacy varied based on the type of intervention and the context of athletic exposure. The results indicated that, overall, injury prevention programs can reduce SRC incidence rates by 34%. This substantial reduction in SRC injury rates represents promising evidence for ongoing efforts to mitigate risks associated with SRC. Furthermore, the preventive efficacy is not influenced by intervention type (physical- vs. educational-based) or the athletic exposure context (practice vs. match).

Compared with other intervention strategies, the efficacy of injury prevention programs identified in this meta-analysis was second only to policy changes. Eliason et al. [13] reported that mouthguard use in collision sports reduced SRC risk by 26%. Disallowing bodychecking in youth ice hockey led to a 58% reduction, and limiting contact during American Football practices reduced SRCs by 64% [13]. Since the majority of SRCs occur as a result of player contact [30], disallowing or limiting contact is arguably the most direct and effective preventive strategy. The preventive effect of mouthguards on SRC remains inconclusive [13, 37]. Although a 26% reduction was observed in a pooled analysis [13], the estimate was heavily influenced by a single study in ice hockey, which accounted for 83% of the total weight [38], potentially limiting the generalizability of the findings. Moreover, another meta-analysis found no significant difference in SRC incidence between athletes who wore mouthguards and those who did not [37], further underscoring the uncertainty surrounding their effectiveness in SRC prevention. Therefore, in addition to policy changes, injury prevention programs represent a practical and effective strategy for reducing the risk of SRC.

Unexpectedly, we found that the efficacy of SRC injury prevention programs in reducing SRC is not influenced by the type of intervention. Notably, our finidings demonstrated a 39% reduction in SRC incidence rate associated with physical training-based injury prevention programs (IRR = 0.61, 95% CI 0.41 to 0.89), although the reduction was not statistically significant. Physical training-based injury prevention programs implemented in the included studies could be categorized into multicomponent exercise programs [20–22, 36] and neck muscle training programs [17, 18] (Table 6). Multicomponent exercise programs encompass a variety of exercises aimed at enhancing different aspects of physical ability (e.g., strength, power, or balance) and are typically implemented during pre-game warm-ups [20–22, 36]. Conversely, neck muscle training programs specifically target the neck musculature and are often incorporated into regular training routines [17, 18].

**Table 6:** Components of physical training

Study		Balance/dynamic stability	Plyometric	Resistance	Landing/cutting technique	Running	Core stability	Sport-related games
Attwood et al. (2018) [20]	Multicomponent exercise program	√	√	√*	√	√		
Barden et al. (2022) [21]		√	√	√*	√			
Hislop et al. (2017) [22]		√	√	√*	√			
Obërtinca (2024) [36]		√	√	√	√	√	√	√
Gillies et al. (2022) [17]	Neck muscle training program			√*				
Peek et al. (2023) [18]				√*				

*Included neck strength training

The observed reduction in SRC incidence rate following physical training interventions may be attributed to improvements in neck strength and balance/dynamic stability. In the six studies with physical training-based programs included in our review, five incorporated neck strength training (Table 6). SRCs can result from a direct blow to the head or body contact that transmits inertial impulse forces through the neck to the head [6, 39]. Enhanced neck strength may mitigate the risk of SRC by improving stability between the trunk, neck, and head [40]. This improved coupling helps stabilize the head during impacts, such as head-on-body collisions in rugby or head-to-head impacts in soccer [40]. Enhanced stability may also aid in dissipating the impact forces transmitted to the brain, thereby reducing the risk of SRC [22]. In fact, athletes with greater neck strength experience lower magnitudes of head impacts in team sports [40]. Moreover, in male professional rugby players, low neck extension strength has been identified as a risk factor for SRCs, with a 10% increase in neck extensor strength associated with a 13% decrease in the incidence rate of SRCs [11]. Integrating neuromuscular neck exercises into the FIFA 11 + program has been also shown to reduce head acceleration during headers and lower the risk of diagnosed SRCs among adolescent soccer players [18, 41]. Neck muscle training is likely a critical component contributing to the efficacy of physical training-based programs in reducing SRC rates. In addition, balance and dynamic stability training appear to be also vital for SRC prevention. Poor balance can impair a player’s motor control and body awareness, leading to suboptimal tackle techniques and an increased risk of SRC [42]. For instance, a previous study reported that rugby players exhibiting poor dynamic balance have a threefold higher risk of sustaining a SRC, even when accounting for concussion history [42]. Given the established connection between poor dynamic balance and a higher risk of SRC, alongside the fact that four out of six physical training-based studies included balance or dynamic stability exercises, it is crucial to incorporate such training into SRC prevention programs.

Concussion safety education commonly aims to equip athletes with the knowledge and skills necessary to effectively manage situations that might lead to SRCs. According to our findings, educational-based injury prevention programs implemented in two of the included studies can reduce the SRC incidence rates by 24%, although this reduction was not statistically significant (IRR 0.76; 95% CI 0.45 to 1.30). Notably, both included studies implemented the HUF program [24, 26], an intervention focused on comprehensive coach education and contact training designed to enhance the safety of American Football players during practice and matches [13, 26]. However, two studies, in fact, yielded conflicting results. Shanley et al. reported that the HUF program reduced SRC incidence rate during practices by 47% (IRR 0.53; 95% CI 0.31 to 0.93)) [24], while Kerr et al. found no significant difference in SRC injury rates following HUF program implementation during either practices or matches [26]. A key distinction between the two studies was the implementation of a fidelity audit schedule in Shanley et al.'s study [24], which was absent in Kerr et al.'s study [26]. The fidelity audit ensured that schools participating in the study were assessed for compliance with heads-up techniques during all contact drills in at least three monitored practice sessions [24]. Previous research has emphasized the critical role of supervision in ensuring adherence to safety protocols [25]. High school teams with a player safety coach overseeing compliance with HUF protocols experienced lower injury and SRC rates during practice compared to teams that implemented HUF without such oversight [25]. This suggests that while the HUF program is a potentially effective educational-based SRC injury prevention intervention, proper supervision may be essential for ensuring adherence.

Key components of the HUF program related to SRC risk reduction include hands-on training in equipment fitting, strategies to minimize player-to-player contact, and teaching proper tackling techniques [43]. For instance, having thicker padding over the zygoma and mandible area may reduce SRC incidence rates in American Football [44, 45], and inadequate helmet fit can increase SRC rates in ice hockey [46]. Alongside this, player-to-player contact accounts for the majority of SRCs in collegiate and elite-level female soccer (80–91%) [14], with tackling identified as the most common concussive event in both American Football (37–48%) [14] and rugby (58–64%) [11]. Therefore, strategies aimed at reducing player-to-player contact and improving tackling techniques, such as heads-up tackling [47], may also be crucial in reducing SRC incidence. Although instruction on equipment fitting, strategies to reduce player-to-player contact, and proper tackling techniques within the HUF program may contribute to its efficacy in reducing SRC incidence rates, the current review was limited to studies that compared educational-based SRC injury prevention programs with a control group. This eligibility criteria led to the exclusion of a study that compared educational-based interventions with and without a player safety coach [25]. Thus, only two studies (with three data points) were included in the final analysis, both focusing on the HUF program in American Football [24, 26]. Additional research is necessary to evaluate the efficacy of various educational-based interventions in reducing SRCs across different sports.

An unexpected finding of this study is that while SRC injury prevention programs effectively reduce SRC incidence rates, the athletic exposure context did not significantly moderate SRC incidence. These findings do not support our hypothesis, indicating that the efficacy of injury prevention programs in reducing SRC incidence rate was similar in both match and practice context. One possible explanation is that SRC injury prevention programs enhance athletes’ physical abilities and technical skills, enabling athletes to navigate varying situations associated with SRC across different athletic exposure contexts, leading to comparable efficacy in SRC prevention. This finding contrasts with the work of Nuhu et al., who reported the FIFA 11 + warm-up program reducing injuries by 40% during matches and 30% during practice [29]. The discrepancy may stem from differences in injury definitions; our study focused solely on confirmed SRCs, whereas Nuhu et al. defined injury more broadly as any physical complaint resulting from soccer participation, regardless of whether it occurred in practice or a match [29]. Given that match intensity is typically higher than in practices, the high-intensity environment may lead to a greater variety and severity of injuries [28]. A broader injury definition could exaggerate the difference in the efficacy of injury prevention programs between practices and matches, making the intervention program appear more effective during matches [29]. In contrast, in studies with a clear injury definition, such as that by Soligard et al., which defined an injury as one that prevented a player from fully participating in the next scheduled match or training session, the efficacy of the injury prevention program was, in fact, similar across both contexts, with an incidence rate ratio (IRR) of 0.72 (0.52 to 1.00) for matches and 0.68 (0.41 to 1.11) for practices [48]. These discussions highlight the importance of specific injury definitions when evaluating the efficacy of intervention programs. Another potential explanation for this discrepancy may lie in the differences in injury mechanisms. Most SRCs are caused by player contact [30], whereas injuries such as ACL tears often result from non-contact mechanisms [49]. Injury prevention programs targeting lower extremity injuries typically focus on enhancing neuromuscular control, balance, and movement patterns during dynamic tasks [50], and their efficacy may be amplified in higher-intensity match situations. In contrast, for contact-related events, the head impacts that lead to SRCs are unlikely to differ significantly between practice and match, even if their intensity levels vary. Neck strength training is one of the few plausible interventions that may help attenuate head impacts [51]; however, only one study within the context-specific subgroup analysis incorporated neck muscle training [17]. Future research is needed to examine whether injury prevention programs that incorporate neck muscle training can differentially reduce the risk of SRC during practice versus match. This may help explain why injury prevention programs demonstrate greater efficacy in reducing broader sport-related injuries during match settings, yet exhibit similar efficacy for SRC across practice and match settings. Nevertheless, our findings are practically meaningful, indicating that consistently implementing targeted training interventions across both matches and practices likely mitigates SRC risks regardless of athletic exposure context.

### Limitations

This review has some limitations. First, the included studies in our review predominantly focused on male participants, with only one study including both male and female subjects [18], and applying these results to female participants should be done cautiously. Given that female athletes are more susceptible to SRCs than male athletes [52] as well as and exhibit a higher number of symptoms and longer durations of symptoms post-injury [53], more attention and research are needed. Second, the injury prevention programs in the included studies were limited to implementation in three sports: rugby, American Football, and soccer. The educational intervention included in our study was limited to the HUF program, which was specifically designed for American Football. Consequently, our findings may not be generalizable to other sports also with high SRC incidence rates, such as ice hockey and lacrosse [30]. Third, most of the reviewed studies were not randomized controlled trials (RCTs), as reflected by the low mean score of 7. The lack of randomization introduces potential biases and limits the interpretation of the findings. If feasible and ethically permissible, RCTs should be used as they are the most rigorous method for assessing the efficacy of a prevention program [10]. Lastly, among the eight included studies, four explicitly used an intention-to-treat (ITT) approach [20, 22, 26, 36], while the remaining four did not specify whether IRRs were based on ITT or per-protocol (PP) analyses [17, 18, 21, 24], which may have affected the estimated effect sizes. However, based on the study descriptions, three likely followed an ITT approach [17, 18, 24]. Therefore, the consistency in analytic methods and the robustness of the pooled estimates support the reliability of the review’s conclusions.

## Conclusion

SRC injury prevention programs could successfully reduce SRC incidence rates by 34% and the efficacy does not vary whether it is physical training or educational intervention as well as in different exposure context. Given the efficacy of both intervention types, healthcare providers and stakeholders can implement these interventions based on their specific contexts to prevent SRC. Further prospective studies are needed to develop injury prevention protocols specifically for female athletes and to explore factors that may influence the efficacy of these interventions, ultimately enhancing the prevention of SRC across various sports.

## Data Availability

The datasets analyzed in this study are available from the corresponding author upon reasonable request. Some data are not publicly accessible, as they were obtained directly from the authors of the included studies, and their approval is required for further access.

## Abbreviations

SRC: Sport-related concussion
HUF: Heads Up Football
IR: Incidence rate
IRR: Incidence rate ratio
AEs: Athlete exposures
