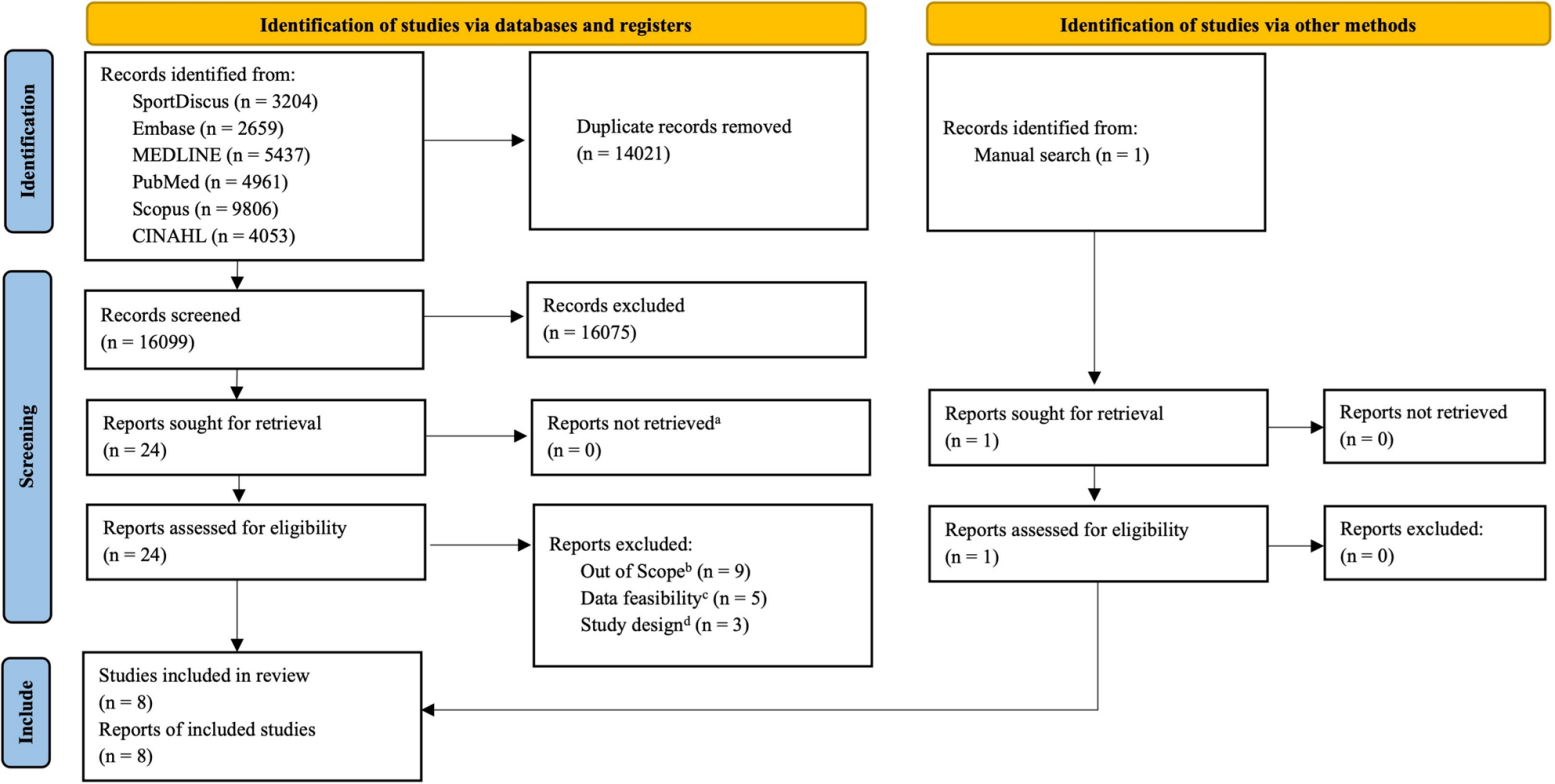# Correction: Sport-related Concussion Can be Prevented by Injury Prevention Program: A Systematic Review and Meta-analysis of Prospective, Controlled Studies

**DOI:** 10.1186/s40798-025-00972-0

**Published:** 2025-12-28

**Authors:** Yan-Long Chen, Tsung-Yeh Chou, Ming-Chih Sung, Yu-Lun Huang

**Affiliations:** 1 Department of Rehabilitation Medicine, Ningbo No. 2 Hospital, Ningbo, China; 2 Faculty of Sports Science, Ningbo University, Ningbo, China; 3 Department of Orthopedic Surgery, University of Texas Health Science Center at Houston, Houston, TX USA; 4 School of Kinesiology, California State University, Angeles, CA USA; 5 Department of Physical Education and Sport Sciences, National Taiwan Normal University, Taipei City, Taiwan

**Correction: Sports Medicine - Open (2025) 11:136**

**https://doi.org/10.1186/s40798-025-00936-4**

The original article presents two errors in Figure 1 whereby the ‘CINAHL (n = 4,053)’ search record is mistakenly omitted and the last box of the study screening flowchart is not displayed in full. The corrected figure can be viewed ahead in this Correction article.